# Validity of self blood pressure measurement in the control of the hypertensive patient: factors involved

**DOI:** 10.1186/s12872-019-1145-9

**Published:** 2019-07-17

**Authors:** Arleen De León-Robert, Juan José Gascón-Cánovas, José Joaquín Antón-Botella, Isabel María Hidalgo-García, Carmen López-Alegría, Yoalys Dilvani Pérez-Cabrera, Heidy Merari Campusano-Castellanos

**Affiliations:** 1 Fortuna Health Centre, Murcia, Spain; 2 Faculty of Medicine (University of Murcia) - Instituto Murciano de Biosanitaria - Arrixaca (IMIB-Arrixaca), Murcia, Spain; 3 Murcia Health Centre, Murcia, Spain; 4 Vistalegre-La Flota Health Centre, Murcia, Spain; 5 Cieza Este Health Centre, Murcia, Spain; 6 Morella Health Centre, Castellón, Spain; 7 Abanilla Health Centre, Murcia, Spain

**Keywords:** Hypertension, Self blood pressure, Primary care, Diagnostic errors

## Abstract

**Background:**

Improving clinical practice aimed at controlling hypertension is a pending issue in health systems. One of the methods currently used for this purpose is self blood pressure measurement (SBPM) whose use increases every day. The aims of this study are to establish the optimal cut-off point for the 3-day SMBP protocol and to identify factors that could affect the precision of the 3-day SMBP protocol using 24-h ambulatory blood pressure monitoring (ABPM) as a reference.

**Method:**

This is a cross-sectional descriptive study to validate a diagnostic test performed by a primary care team in Murcia, Spain. A total of 153 hypertensive patients under 80 years of age who met the inclusion criteria were evaluated. ABPM was performed for 24 h. The SBPM protocol consisted of recording 2 measurements in the morning and 2 at night for 3 days.

**Results:**

The cut-off point for SBP was set at 135 mmHg (sensitivity: 80.39%, specificity: 74.19%), and for DBP, it was set at 83 mmHg (sensitivity: 76.48%, specificity: 84.89%), which yielded the highest combined sensitivity and specificity. After carrying out the validation study with the new figures, we proceeded to establish which socio-demographic factors prevented a correct classification of patients. These errors were more common in male patients for the assessments of both DBP (OR = 2.4) and SBP (OR = 2.5); hypertensive patients with age < 67,5 years (OR = 1,5); having no work activity (OR = 3,6) and with concomitant chronic kidney disease (CKD) (OR = 5.0).

**Conclusion:**

Being male, older than 67.5 years, with CKD or with no work activity increases the probability of being misclassified for hypertension during follow-up as assessed by SBPM over 3 days.

**Trial registration:**

This study was approved by the research ethics committee of the University of Murcia under registration number 1018/2015.

## Background

A primary objective in any primary care consultation is to achieve good blood pressure (BP) control in hypertensive patients in order to reduce the likelihood of future cardiovascular events. Hypertension (HT) in Spain affects 36.7% of the total population, while approximately 40% of the general population in Europe is affected [1]. In both men and women, this prevalence increases significantly with age, reaching 68% of those over 60 year [2]. In terms of morbidity and mortality, HT has been associated with 1 in 2 deaths of cardiovascular origin in individuals older than 50 years [3].

Currently, SBPM is considered a useful tool both in the diagnosis and monitoring of hypertension [1, 4–6]. Although it cannot replace ABPM as a gold-standard assessment method, it is considered a complementary method in the diagnosis of high blood pressure [7].

The main advantage of SBPM lies in its ability to obtain multiple BP records outside of the health care environment. Likewise, SBPM shows considerable agreement with ABPM in regard to detecting normotension and masked hypertension [4]. The SBPM’s correlation with damage to target organs and its ability to predict cardiovascular events is higher than in-office blood pressure measurements [8] and is similar to that of ABPM [9]. Patients monitored with this technique show better adherence to long-term pharmacological treatments and therefore improved control rates, lower follow-up costs, better assessments of the effects of pharmacological treatments at different times of the day, the elimination of observer bias, and less clinical inertia [4].

There is no doubt that SBPM is a useful clinical tool for hypertensive patients in Primary Care, where the good management of the short time available for the consultation and the reliability are essential for appropriate control of HT. Therefore, it is also necessary to have reliable cut-off values ​​and an efficient daily SBPM decision-making scheme. Therefore, after demonstrating that the 3-day protocol is the most efficient option for the control of blood pressure in primary care, with a discriminative capacity and agreement with ABPM similar to other protocols [10, 11], the aims of the present study are to establish the optimal cut-off point for the 3-day SBPM protocol and to identify the socio-demographic factors that can influence erroneous classification by SBPM during the monitoring of hypertensive patients.

## Methods

This is a cross-sectional descriptive study for the validation of a diagnostic test.

### Population

We worked with hypertensive patients registered in the computer system at the Vistalegre-La Flota Urban Health Centre in Murcia, Spain and those receiving routine care.

### Study period

December 2011 to December 2012.

### Criteria for inclusion and exclusion

Inclusion criteria included the diagnosis of complicated and uncomplicated hypertension, an age of 18–80 years, sufficient vision and hearing to perform the self-measurement, and adequate intellectual capacity to obtain the measurements or the oversight of a responsible caregiver for doing so. The exclusion criteria that were considered are those considered valid in various international HT guidelines [1, 5]: Immobilized patients without a responsible caregiver and hypertensive patients diagnosed with obsessive-compulsive disorder.

### Selection mechanism

Duplicates, diagnostic errors, patients in the computerized clinical history, and those with their last visit to the health centre (to see a doctor or nurse) in the year prior to the start of the patient selection (*N* = 2,245) were filtered from anonymized lists. The sample was calculated with an accuracy of 5%, a confidence level of 95%, and a sensitivity and specificity of 85.7 and 75%, respectively. An estimated prevalence of patients with uncontrolled BP of 35% (*n* = 141) was determined. From this figure, we calculated a percentage of expected losses of 15%, leading to a sample size of *n* = 153. The calculation of the sample size was performed using the online calculator by Fisterra [12].

### Sampling method

**Fig. 1:**
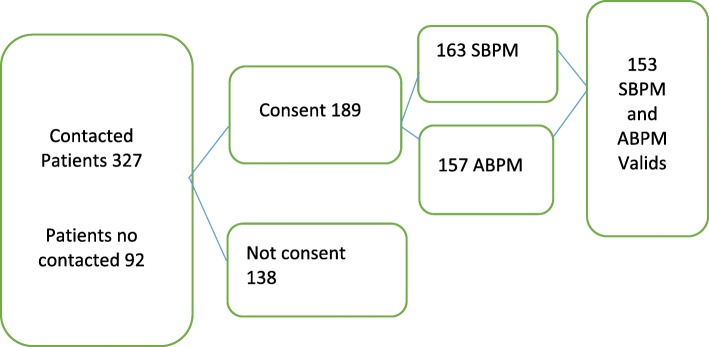
Flow chart diagram of patient inclusion

A systematic random sampling procedure was carried out. The first subject was chosen at random, and the sampling fraction used was 1/10 patients. The patients were recruited through telephone contact or through their doctors, for those who went to the health centre during the patient selection period. In the case of negative or no contact after several attempts, the next patient was chosen from the list. The recruitment flowchart is shown in Fig. 1.

After giving consent, each selected patient who agreed to participate was scheduled for an appointment at 8:30 a.m. to perform the ABPM. The perimeter of the patient’s arm was measured, and the appropriate cuff was provided. If the arm circumference was greater than 32 cm, a large cuff was provided. The blood pressure was measured in both arms, and the non-dominant arm was chosen as the measuring arm. If both measurements were equal, the cuff was placed on the left arm for right-handed patients or on the right arm for left-handed patients. After the placement of the ABPM device, a forced measurement was immediately carried out. The recording began in the morning at the time the device was placed.

The patients were instructed to perform normal daily activities, except that when the cuff warned that a measurement was about to begin, the arm should be kept in a relaxed position. The patients were asked to return the next day at the same time.

The programming of the device was as follows: Frequency of readings: every 15 min during daytime and every 30 min while sleeping. For this, the patient was questioned when placing the device about their sleep schedule, which was confirmed and readjusted the next day when the device was removed. With these data, the program calculated the beginning of the night or sleep period and the day or activity period for the purposes of the analysis. Types of recordings: Measurement of SBP, DBP and HR over 24 h, daytime and evening. Measurement range: HR: 40 to 180 beats per minute. Pressure: 70 to 285 mmHg for systolic; 40 to 200 mmHg for diastolic and 60 to 240 mmHg for mean blood pressure values. Valid record criteria: 70% valid measurements on the ABPM, more than 14 valid measurements of systolic and diastolic BPs during the day and more than 7 measurements of systolic and diastolic BPs during the night.

The next day, the ABPM device was removed and the patient was shown how to use the validated semi-automatic tensiometer to obtain SBPM records. The subject was seated, and after a period of 5 min of rest, 2 measurements were taken from the dominant arm with a 1-min interval between them. A third measurement was taken if the first 2 had a difference of greater than 5 mmHg.

After the demonstration, the patient was instructed in the proper handling of the device, specifically that the measurements should be taken while sitting, at rest, with the cuff placed on the arm that showed the highest BP. The patient was also instructed on how to place the cuff, which was to be 2–3 cm above the flexure of the elbow, its fit on the arm, and the position at which to take the pressure reading.

The correct performance of the first 2 measurements on the first day was verified by the patient in front of the researcher. Then, the blood pressure monitor was given to the patient, who was instructed in writing to take 2 measurements in the morning (between 7:00 and 10:00 a.m.) and 2 before going to bed (between 21:00 and 23:00 p.m.) with 1–2 min between measurements for 3 consecutive days. A total of 12 measurements were obtained for each patient (the first day measurements were later discarded), establishing the 3-day SBPM pattern. The instruments used in this study included 2 ABPM devices *Microlife Watch BP 03 (Microlife, Widnau, Switzerland)* [13] and 10 automatic arm blood pressure monitors for SMBP (*Microlife Watchbp Home)* that were validated according to the standards of the Spanish Hypertension Society (SEH) and the British Hypertension Society (BHSOC) (accessible on January 11, 2016 at: https://www.seh-lelha.org/microlife-watchbp-o3/ and https://bihsoc.org/bp-monitors/for-home-use/).

Regarding the definitions of the variables applied in this study, we defined poor HT control as when the mean BP measured by ABPM over 24 h was greater than 130/80 mmHg. For the 3-day SMBP, we considered uncontrolled average blood pressure as greater than 135/85 mmHg.

This descriptive study was carried out with the demographic variables of age, sex and work status, taking into account the records of spanish administrative document (TSI) that accredits access to citizens to health care benefits. If the patient was not active: TSI < 002 (Without income, retired) or active: TSI 003 (income level < € 18,000/year); TSI > 4 (income level > € 18,000/year). Clinical variables included associated comorbidities such as a diagnoses of dyslipidaemia, diabetes mellitus, chronic kidney disease, atrial fibrillation, and stroke.

### Statistical analysis

The statistical programs SPSS version 22 and Epidat version 3.1 were used to analyse the data.

Categorical variables are presented as absolute frequencies (%) and quantitative variables as the means and standard deviations. Values of *p* < 0.05 were considered statistically significant, and 95% confidence intervals (95%CI) were calculated. The sources of the data were the BP records collected by SBPM and ABPM as well as the electronic health records of the patients in the OMI-AP program.

### Test validation analysis

A comparison of the data collected using the 3-day SBPM (discarding measurements from the first day) was performed using the figures obtained by the 24-h ABPM as the reference standard. Given the results obtained by De León-Robert et al. [10], the 3-day pattern was chosen (SBPM-DAYS-2&3) and an optimal cut-off point was obtained. The calculations of the predictive capacity of the 3-day protocol with the new cut-off point were performed using a statistical calculator. With the data obtained, the cases with misclassifications (false positives and negatives) in the SBPM-DAYS-2&3 data were identified at the cut-off point where the sensitivity and specificity of the diagnosis of HT were optimized, using the 24-h ABPM as the gold standard (130/80 mmHg). An analysis of how sociodemographic and clinical factors related to errors in diagnostic classifications was performed. Calculations were performed for the crude and adjusted odds ratios, according to sex, of the diagnostic classification errors for both systolic and diastolic HT. These were performed using logistic regression models that included age, income level, and clinical comorbidities such as diabetes, dyslipidaemia, chronic kidney disease, ictus, and atrial fibrillation as predictor variables. The estimating of the agreement between ABPM and SBPM readings were perfomed using the Bland-Altman plot analysis and the concordance was determinated using intraclass correlation coefficient (ICC).

## Results

A total of 419 patients were selected for inclusion in the analysis, of whom 153 completed SBPM and ABPM with the required quality (Fig. 1). Of these patients, 50.3% were women. The mean age was 61.54 with a range of 23–80 years. In terms of economic activity, 67.3% were classified as having no work activity (pensioners and the unemployed) (Table 1).

**Table 1:** Socio-demographic and baseline clinical characteristics of the study subjects

Gender	n	%
Male	76	49.7
Female	77	50.3
Age group		
(23–57.5 years)	50	32.7
(57.51–67.5 years)	52	34.0
(> 67.5 years)	51	33.3
TSI		
Inactive (TSI 1 Unemployed; TSI 2 Retired)	103	67.3
Active (TSI 3 Income below € 18,000/year; TSI 4 Income above € 18,000/year)	50	32.7
Comorbidities		
Diabetes	41	26.8
Chronic kidney disease	13	8.5
Dyslipidaemia	64	41.8
Atrial fibrillation	7	4.6

n: absolute frequency. %: relative frequency

In addition to HT, other associated chronic pathologies were found among the hypertensive patients studied (Table 1). The most common was dyslipidaemia (41.8%).

**Fig. 2:**
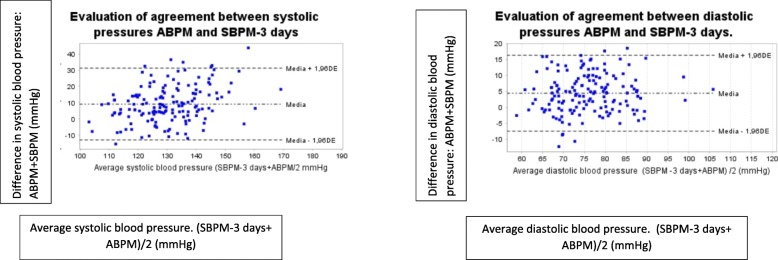
Bland-Altman plots. SBPM-3 days: Self-blood pressure monitoring during 3 days. ABPM: 24H Ambulatory blood pressure monitoring

According to the results of the ROC curve, the cut-off point where sensitivity and specificity were optimized was 135.5/83 mmHg for the diagnosis of uncontrolled HT. The mean differences between the two methods (ABPM and SBPM) are 10 and 5 mmHg for systolic and diastolic pressure respectively (Fig. 2). The concordance between SBPM and ABPM according ICC was higher for diastolyc pressure (0,64; *p* < 0.001) than for systolic pressure (0,44; *p* < 0.001).

Based on this cut-off point, the sensitivity of the SBPM-DAYS-2&3 test for identifying cases with control problems in some BP measurements was 87.7%, while its specificity was notably lower (62.5%) (Table 2). More specifically, this test was more sensitive for the isolated diagnosis of abnormal ​​SBP values (87.7%) than for diagnosing abnormal DBP values (70.6%); In contrast, it had a greater specificity when the uncontrolled blood pressure was diastolic (85.7%) instead of systolic (74.2%) (Table 2). On the other hand, the predictive capacity of the test for the diagnosis of a lack of control of systolic or diastolic values, with a prevalence in the study sample of 42.5%, was elevated for a negative result (87.3%) and moderate for a positive result (63.35%) (Table 2).

**Table 2:** Validation of self-monitoring of blood pressure for 3 days (cut-off point = 135.5/83 mmHg) for the control of hypertension. Gold standard: 24-h ambulatory blood pressure monitoring

	Systolic hypertension			Diastolic hypertension			Joint HT		
	Ep	95%CI		Ep	95%CI		Ep	95%CI	
Sensitivity (%)	80.4	69.1	91.7	70.6	53.8	87.3	87.7	79.0	96.4
Specificity (%)	74.2	65.0	83.4	85.7	79.0	92.4	62.5	51.8	73.2
Positive predictive value (%)	64.3	52.4	76.2	58.5	42.2	74.8	63.3	52.8	73.8
Negative predictive value (%)	86.7	78.8	94.6	91.1	85.3	96.8	87.3	78.3	96.3
Positive likelihood ratio	3.1	2.2	6.5	4.9	1.7	4.9	2.3	2.6	9.91
Negative likelihood ratio	0.3	0.22	0.46	0.3	0.1	0.3	0.2	0.3	0.6

Prevalence of uncontrolled status: 42.5%

*Ep* point estimate, *HT* hypertension, *%* percentage, *95% CI* 95% confidence interval

With the SBPM-DAYS-2&3, approximately 1 out of every 4 hypertensive subjects had their systolic HT incorrectly classified (false positive or negative); this figure dropped to 1 out of every 6 for the assessment of diastolic HT. The variables associated with obtaining an erroneous result with the SBPM-DAYS-2&3 test were sex, age, work status and chronic kidney disease. These errors were approximately 2.5 times more common in male patients for the assessments of both DBP (OR = 2.4) and SBP (OR = 2.5). Regarding the latter, in subjects of more advanced ages (> 67.5 years), the validity of the test was lower (OR = 1.5). The same pattern occurred in patients with TSI < 3 (low income level) (OR = 3.6) and in hypertensive patients with concomitant chronic kidney disease (OR = 5.0) (Table 3).

**Table 3:** Factors that influence the misclassification of hypertension by SBPM. Gold standard: ABPM (130/80 mmHg). SBPM test 1 on 3rd day: (135.5/83 mmHg)

	SMBP SBP		SMBP DBP	
	OR (95%CI)	Adjusted OR^a^	OR (95%CI)	Adjusted OR^a^
Male	2.1 (0.99–4.5)	2.5 (1.1–5.5)^*^	2.3 (1.0–5.5)	2.4 (1.0–5.9)^*^
Age > 67.5 years	1.5 (1.0–2.2)^*^	1.5 (1.0–2.3)^*^	1.2 (0.8–1.9)	1.2 (0.8–1.9)
TSI < 3	2.9 (0.8–10.0)	3.6 (1.0–12.5)^*^	1.4 (0.5–3.9)	1.2 (0.4–3.4)
Diabetes	1.3 (0.6–2.8)	2.1 (1–4.5)	1.1 (0.9–2.1)	1.1 (0.9–2.3)
IRC	6.4 (2–21.2)^*^	5 (1.9–20)^*^	2.3 (0.9–8.3)	1.9 (0.9–8.3)
Dyslipidaemia	1.1 (0.9–1.8)	1.1 (0.9–1.9)	1.1 (0.9–2)	1.1 (0.9–2)
Fibrillation	1.3 (0.9–7.1)	1.1 (0.9–5.5)	1.3 (0.9–11.2)	1.8 (0.9–15.6)

*HT* High blood pressure, *SBPM* Self-monitoring of blood pressure, *ABPM* Ambulatory monitoring of blood pressure, *SBP* Systolic blood pressure, *DBP* Diastolic blood pressure, *OR* Odds ratio, *TSI < 4* unemployed, retired, income less than € 18,000, *95% CI* 95% confidence interval, *CKD* Chronic kidney disease ^*^ *p* < 0.05. ^**^ *p* < 0.01.^a^Adjusted for sex by logistic regression using the ENTER method

## Discussion

The diagnostic capacity of SBPM, using ABPM as a reference, for the assessment of HT has been widely investigated by several studies [14]. The possibility of using its different protocols without diminishing that capacity has also been investigated, with good results [15]. Taking into account the data obtained to date, as well as the need in primary care for an efficient protocol, we decided to evaluate the SBPM-DAYS-2&3. We also investigated which factors we should take into account for its correct application and interpretation. The analysis with ROC curves for the 3-day protocol resulted in cut-off values of 135.5 mmHg for SBP and 83 mmHg for DBP, differing from the results of another study [11] by 3 mmHg for SBP and just 0.2 mmHg for DBP. However, the SBPM-DAYS-2&3 yields higher values for both sensitivity and specificity for both pressures with our cut-off values.

The current guidelines on hypertension by various societies do not provide clear recommendations in their protocols about all of the factors that can diminish the accuracy of SBPM as a follow-up method in HT. The Japanese and Brazilian guidelines refer to the need for bladder emptying before taking BP measurements, something not mentioned in the European or American guidelines [16]. For Almeida et al. [16], who also used the 3-day protocol, a factor that limits the accuracy of SBPM is whether BP measurements are taken after urination. Other studies, such as ours, note that sex and age are possible factors. However, they also add the systolic and diastolic figures, the number of readings, food intake, alcohol consumption, the taking of BP measurements after bathing, and being a smoker [14, 17–19]. However, some of those studies accounted for the daytime ABPM to make their comparisons [16, 19].

Among the clinical factors evaluated by our study, it is worth mentioning the significant influence of the diagnosis of chronic kidney disease in the sample as a factor involved in limiting the accuracy of the SBPM-DAYS-2&3 protocol. Studies in kidney patients indicate greater arterial rigidity in these patients, which could be the cause. Although arteriosclerosis is also common among diabetic patients, no significant difference was found between diabetic and non-diabetic patients.

### Limitations of the study

This study was performed with a small sample size in a single health centre in Spain, wich is not necessarily representative of the general population. However, as a strength, the study was conducted in the field of usual clinical practice.

## Conclusions

The cut-off point with the highest joint sensitivity and specificity for the SMBP-DAYS-2&3 protocol is 135.5/83 mmHg. Being male, over 67.5 years of age, having low income level, and having a diagnosis of chronic kidney disease can all reduce the validity of the SMBP-DAYS-2&3 protocol for classifying hypertensive patients as poorly controlled during follow-up.

## Data Availability

The datasets generated and/or analysed during the current study are not publicly available due to data privacy laws but are available from the corresponding author on reasonable request.

## Abbreviations

ABPM: Ambulatory blood pressure monitoring for 24 h
DBP: Diastolic blood pressure
HR: Heart rate
HT: Hypertension
IC95%: 95% confidence interval
ROC CURVES: Receiver operating characteristic curves.
SBP: Systolic blood pressure
SMBP: Self-monitoring of blood pressure
SMBP-DAYS-2&3: 3-day protocol of self-monitoring of blood pressure
TSI: Individual health card
